# Immunometabolic reprogramming of macrophages and neutrophils in acute pancreatitis: from metabolic stress to amplification and endogenous braking

**DOI:** 10.3389/fimmu.2026.1976931

**Published:** 2026-10-13

**Authors:** Yuanhang Ai, Longze Zhang, Aiping Wei, Jiayan Dong, Tianjing Pang, Yuanyu Zhang, Kaifeng Wu, Shifei Yao

**Affiliations:** 1 Department of Laboratory Medicine, The Third Affiliated Hospital of Zunyi Medical University (The First People’s Hospital of Zunyi), Zunyi, China; 2 Scientific Research Center, The Third Affiliated Hospital of Zunyi Medical University (The First People’s Hospital of Zunyi), Zunyi, China

**Keywords:** acute pancreatitis, immunometabolism, macrophage, metabolic reprogramming, metabolic stress, neutrophil

## Abstract

Acute pancreatitis (AP) progression involves both the initiating acinar injury and immune responses that sustain or contain subsequent damage. This review integrates evidence on metabolic regulation in macrophages and neutrophils to examine its links to inflammatory effectors, clearance, and tissue protection. Glycolytic regulation can support inflammatory activation, while non-AP neutrophil studies show how substrate allocation supports oxidant production and neutrophil extracellular trap (NET) release. Metabolite signaling also participates in macrophage efferocytosis, repair-associated responses, and tissue antioxidant defense. Contrasting lactate effects in acinar injury and macrophage repair indicate that metabolic changes must be interpreted through their effects on particular cells and functions. We propose an integrated amplification-braking framework that connects metabolically supported inflammatory effectors to continued generation of injury-derived stimuli, and clearance, antioxidant, and repair functions to damage containment. These functions can coexist without receiving equivalent metabolic support, and protective braking is distinct from generalized immunosuppression. Evidence differs across cells and experimental systems; metabolic regulation of inflammatory activation does not by itself establish metabolic control of NET-mediated injury in AP. The framework provides a basis for evaluating whether metabolic interventions can limit damaging activity while preserving protective functions, but it is not a validated clinical model. Pairing metabolic changes with specific cellular functions and subsequent injury is central to testing these relationships.

## 1 Introduction

Acute pancreatitis (AP) is one of the most common gastrointestinal emergencies worldwide, with an estimated annual incidence of approximately 34 cases per 100,000 people and a steadily increasing hospitalization burden (1). Clinically, its presentation spans a broad spectrum, ranging from mild, self-limiting local inflammation to life-threatening disease characterized by pancreatic necrosis, persistent organ failure, and infectious complications (2–4). The initiating acinar injury alone does not explain this divergence. Macrophage and neutrophil responses can aggravate inflammation and acinar damage, while macrophage-mediated clearance and repair contribute to recovery (5–7). Systemic inflammatory response syndrome (SIRS) and compensatory anti-inflammatory response syndrome (CARS)-associated responses can coexist in experimental AP (8). Understanding disease persistence therefore requires an explanation of both immune activation and the adequacy of functions that contain tissue damage.

Immunometabolism provides a mechanistic basis for examining these functional differences. Substrate use, metabolic enzyme regulation, and metabolite signaling influence immune-cell production of inflammatory mediators, responses to oxidative stress, and clearance of apoptotic cells (9–11). AP-related studies connect glycolytic regulation in macrophages and neutrophils to inflammatory activation (12, 13), while metabolite studies connect metabolic regulation to macrophage repair, efferocytosis, and tissue antioxidant protection (14–16). These observations point to the possibility that metabolic reprogramming of immune cells may influence AP progression by shaping both inflammatory activity and the protective functions that limit tissue damage.

Recent AP reviews have clarified several parts of this biology. Some have focused on systemic lipid and glucose metabolism (17), oxidative stress and damage-associated molecular pattern (DAMP) immune interactions (18), macrophage functional states (19), and the intersection of ferroptosis, metabolism, and immunity (20). Here, we ask how metabolic support for distinct macrophage and neutrophil functions shapes the persistence or containment of pancreatic injury. Examining inflammatory effectors alongside clearance and antioxidant functions may explain how new injury is sustained or injury-derived stimulation is curtailed, without assigning a fixed damaging or protective role to a cell state or metabolic pathway.

Here, we propose an integrated amplification-braking framework to examine how macrophage and neutrophil immunometabolism contributes to the persistence or containment of pancreatic injury. To synthesize the current immunometabolic landscape in AP, we conducted a literature search across PubMed and Web of Science covering publications up to August 2026. Search terms combined “acute pancreatitis” with terms related to macrophages, neutrophils, metabolic stress, metabolic pathways, and immunometabolites. Priority was given to recent studies demonstrating direct functional or metabolic phenotypes in AP patients or experimental models, while foundational non-AP immunometabolic mechanisms were cited to support extrapolated hypotheses within our proposed framework.

## 2 Metabolic stress microenvironment in AP

In acute pancreatitis (AP), macrophages and neutrophils sense tissue danger signals in a changing environment that lacks oxygen and stable nutrient supply. Acinar injury releases intracellular material, lipolysis and lipid oxidation alter local lipid composition, and impaired perfusion can restrict oxygen delivery. Changes in intestinal barrier function and microbial metabolism provide additional inflammatory stimuli and regulatory metabolites. Their combination varies with etiology and injury severity rather than following a fixed sequence shared by all forms of AP. **Figure 1** summarizes these input categories.

**Figure 1:**
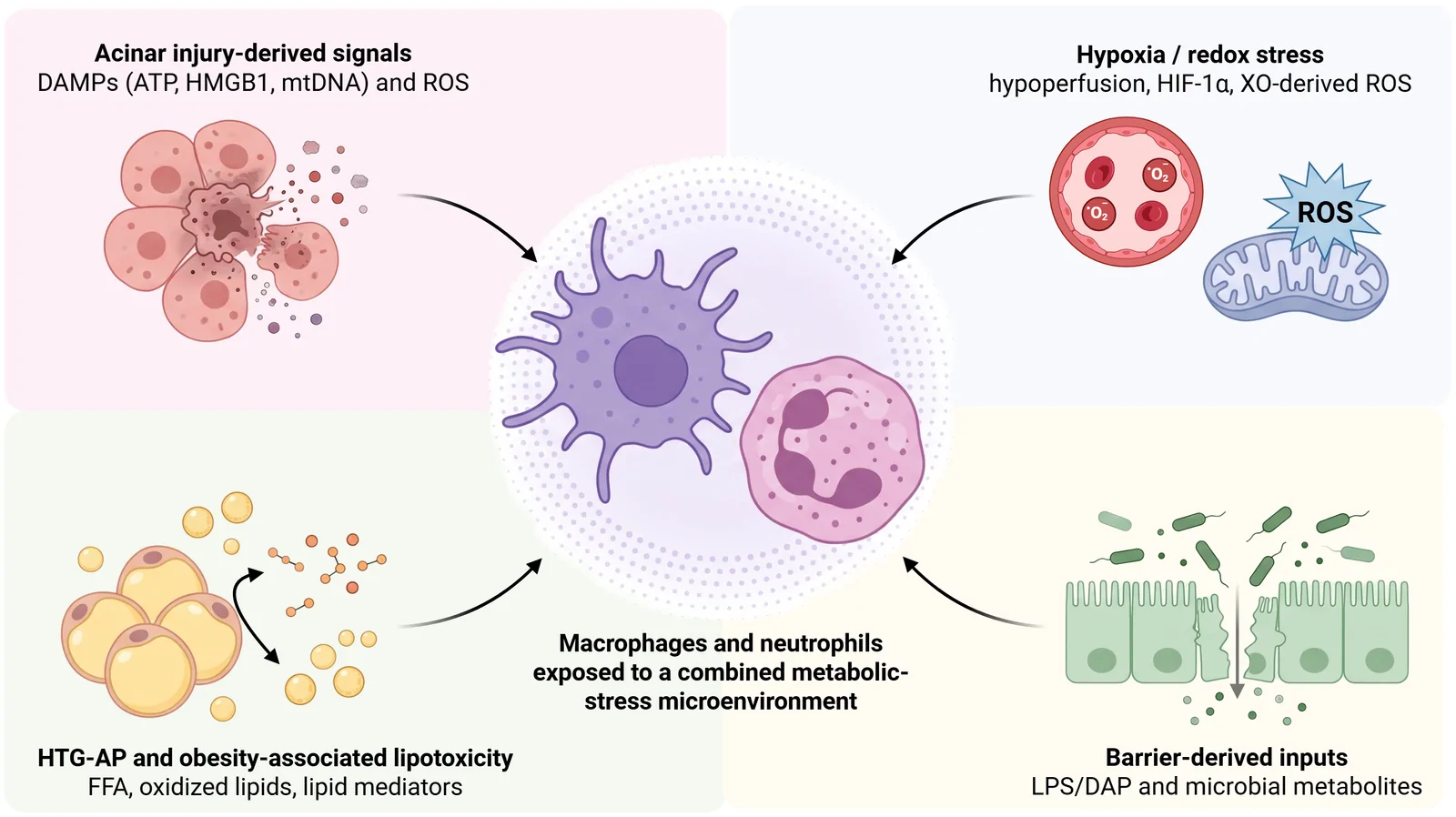
Metabolic stress microenvironment encountered by macrophages and neutrophils in acute pancreatitis. Acinar injury-derived danger signals, HTG-AP/obesity-associated lipotoxicity, hypoxia/redox stress, and barrier-derived microbial inputs together shape the exposure environment of macrophages and neutrophils in AP. These inputs differ in source, timing, and disease context, but converge on myeloid cells and provide the upstream context for later metabolic and functional reprogramming. AP, acute pancreatitis; HTG-AP, hypertriglyceridemia-induced acute pancreatitis; DAMPs, damage-associated molecular patterns; FFA, free fatty acids; ROS, reactive oxygen species; DAP, diaminopimelic acid. Created with Biorender.com.

### 2.1 Acinar injury-derived danger signals and mitochondrial stress

Metabolic collapse in acinar cells triggers tissue injury, thereby releasing danger signals that activate the immune response. Abnormal calcium signaling, mitochondrial dysfunction, and intracellular digestive enzyme activation are interrelated processes in early acinar damage (21). Impaired mitochondrial energy supply compromises cellular homeostasis; when injury releases intracellular contents, ATP, DNA, and high-mobility group **Box 1** (HMGB1) become DAMPs (22). In cerulein-induced AP, studies of Toll-like receptor 9 (TLR9) and P2X7 signaling support a role for DNA and extracellular ATP as danger signals that promote inflammation, while experiments targeting the NLR family pyrin domain-containing 3 (NLRP3) inflammasome implicate downstream inflammatory signaling in pancreatic injury (23). Release of these molecules provides a route by which acinar energy failure can stimulate immune activation.

Box 1 **Candidate paired readouts for amplification and braking in AP.**

**Table:** 

Function or context	Candidate readouts, methods, and samples	Interpretation and evidence status
Injury and inflammatory context	Blood CRP and leukocyte counts; organ dysfunction and clinically obtained assessments of pancreatic necrosis; pancreatic histology in animals.	Describe disease course and outcomes rather than immunometabolic amplification itself. Compare functional readouts at similar initial injury severity.
Amplification-related effectors	Circulating IL-1β and NET-associated citrullinated histone H3 or MPO–DNA; imaging of extracellular DNA colocalized with neutrophil proteins in isolated cells or animal pancreas. Pair with neutrophil metabolic measurements and, experimentally, acinar injury.	AP studies support examining NET-associated injury (6, 76, 91). MPO–DNA assays in human plasma require specificity controls, including isotype controls; plasma measurements alone cannot establish tissue NETs (106). Pair with complementary imaging, cellular metabolism and injury; a metabolic cause requires perturbation.
Braking-related clearance	Flow cytometry or imaging of labeled apoptotic-cell uptake by macrophages; macrophage/apoptotic-cell costaining and uncleared apoptotic cells in animal pancreas.	Functional uptake has experimental AP support (15, 54). Distinguish internalization from surface binding. Patient-derived phagocyte assays are proposed extensions; Anxa1 or PPARα expression cannot substitute for clearance function.
Braking-related antioxidant protection	GPX4 protein measurements paired with lipid-peroxidation assays and tissue injury in animal pancreas or experimental cells; pancreatic TBARS and histology were used in the ACOD1 study (16).	AP evidence links ACOD1-associated protection to reduced oxidative injury (16, 67). Protein abundance alone does not establish antioxidant activity. TBARS lacks cell specificity and does not independently establish ferroptosis.
Metabolism supporting either function	ECAR/OCR in isolated monocytes or neutrophils under defined stimulation; ACOD1 and related transcripts in specified cells; targeted mass spectrometry of NorCA or itaconate in validated sample types. Stable-isotope tracing can test glycolytic or PPP flux in experimental cells.	Selected bioenergetic and metabolite measurements have AP support (15, 56); pathway evidence also comes from the ACOD1 study (16). Expression, concentration and ECAR are not pathway-specific flux. Stimulated neutrophil OCR includes non-mitochondrial oxygen consumption. Pancreatic itaconate was not directly quantified in Ref. 16; PPP tracing is a proposed AP extension of non-AP work (60).
Immune responsiveness and host defense	Flow-cytometric HLA-DR mean fluorescence intensity on CD14hiCD16− monocytes in isolated blood PBMCs; ex vivo restimulation and infection follow-up as proposed complements.	AP cohort evidence includes repeat measurements in selected patients (107), but does not validate this combined assessment. Low HLA-DR alone establishes neither monocyte suppressive function nor effective clearance or antioxidant protection.

AP, acute pancreatitis; CRP, C-reactive protein; MPO, myeloperoxidase; NETs, neutrophil extracellular traps; TBARS, thiobarbituric acid-reactive substances; ECAR, extracellular acidification rate; OCR, oxygen consumption rate; PPP, pentose phosphate pathway; NorCA, norcholic acid. Other abbreviations follow the main text. PBMCs, peripheral blood mononuclear cells.

DNA sensing illustrates this route. Supernatants from dying acinar cells activated Stimulator of interferon genes (STING) signaling in bone marrow-derived macrophages, and DNase I treatment attenuated the response. Bone marrow chimera experiments also supported a contribution of hematopoietic STING to experimental AP injury (24). Cellular material released by dying acinar cells can thus stimulate macrophage inflammatory responses, linking the initial acinar injury to further inflammation within the pancreas.

### 2.2 Lipotoxic substrate pressure in HTG-AP and obesity

Hypertriglyceridemia and obesity increase lipid exposure during AP, but circulating triglyceride concentrations alone do not capture the damaging effects of lipids within the pancreas and surrounding adipose tissue. Local lipolysis releases free fatty acids, while tissue injury alters the chemical composition of the lipid pool (25–27). The resulting environment can influence macrophage inflammatory activity as well as the susceptibility of exposed cells to oxidative injury.

Lipid modification can sustain inflammation by interfering with anti-inflammatory responses or stimulating cytokine production. Lipid extracts from AP-associated ascitic fluid contained oxidized fatty acids and weakened the suppression of macrophage inflammation by peroxisome proliferator-activated receptor γ (PPARγ) agonists (26). Fatty acid chlorohydrins released from necrotic fat also increased inflammatory cytokine expression in peritoneal macrophages in experimental AP. Lipid exposure can additionally promote injury through macrophage acinar cell interactions. Palmitate-stimulated macrophages released cathepsin S-containing exosomes that promoted acinar pyroptosis (28), linking fatty acid exposure to an immune-mediated mechanism of pancreatic damage.

Cellular lipid handling and mitochondrial injury provide further links between lipid stress and AP severity. In obesity-associated SAP, CD36 inhibition reduced lipid peroxidation and ferroptosis-associated changes in pancreatic and adipose tissues; complementary experiments in palmitate- and LPS-stimulated macrophages supported a contribution of CD36 to ferroptotic injury and inflammatory responses (29). In experimental HTG-AP, reduced abundance of pancreatic proteins associated with oxidative phosphorylation accompanied mitochondrial structural damage, and procyanidin capsule treatment attenuated these changes and pancreatic injury (30). Together, these findings suggest that lipid excess and modification can aggravate AP both by stimulating macrophage inflammatory activity and by increasing cellular vulnerability to oxidative damage.

### 2.3 Perfusion, redox stress, and HIF-1α signaling

Microcirculatory disturbances during AP can restrict pancreatic oxygen delivery, although the extent of oxygen limitation varies with disease severity (31, 32). In a porcine study, pancreatic perfusion and oxygen tension decreased in severe taurocholate-induced pancreatitis, whereas oxygen tension increased in the mild group receiving intraductal saline (33). Reduced oxygen availability can constrain mitochondrial oxidative metabolism. HIF-1α-dependent transcription provides a means of adapting to this constraint by increasing glycolytic capacity, and non-AP macrophage studies link this metabolic response to inflammatory activation (34–36). These mechanisms suggest that poorly perfused pancreatic regions may favor glycolytic support of immune-cell inflammatory activity, although this connection has not been established by tissue oxygen measurements alone.

HIF-1α also links cellular redox and metabolic signals to inflammation. In experimental necrotizing AP, XO inhibition reduced pancreatic HIF-1α, LDHA, and NLRP3-related responses. Complementary glucose isotope tracing in hypoxic primary acinar cells showed reduced labeled lactate accumulation after XO inhibition, connecting XO activity to altered glucose metabolism in acinar cells (37). In non-AP macrophages, succinate accumulation promoted HIF-1α-dependent IL-1β expression (38). These findings identify routes through which oxygen limitation and intracellular metabolic changes can influence inflammatory responses in different cell populations. In AP, such responses may connect metabolic stress in injured acinar cells with inflammatory activity in recruited macrophages, helping sustain inflammation in poorly perfused tissue.

### 2.4 Gut-derived microbial products and metabolites

Intestinal barrier dysfunction and microbial metabolism provide two routes through which the gut can influence pancreatic inflammation. A clinical study identified increased intestinal permeability and disrupted tight-junction structure within the first two weeks of AP (39). In SAP rats, increased circulating diaminopimelic acid (DAP) accompanied activation of intestinal and pancreatic NOD1/RIP2/NF-κB signaling, and interventions targeting the microbiota reduced both responses (40). Separate experimental work linked the gut microbiota to NLRP3 inflammasome activation and AP severity (41). Impaired barrier integrity thus heightens host exposure to microbial products, driving inflammatory cascades that extend far beyond the initial acinar injury.

Microbial metabolites can also support barrier integrity and restrain inflammatory responses. Short-chain fatty acids (SCFAs) regulate host immune function through receptor-mediated signaling and other mechanisms (42, 43). In experimental AP, Bifidobacterium pseudolongum-derived acetate suppressed macrophage inflammatory responses through GPR43/FFAR2-associated signaling (44), while butyrate supplementation and dietary inulin improved intestinal barrier measures and reduced pancreatic injury in their respective models (45). Protection varied across models, indicating that these metabolites do not have interchangeable effects (46). Several interventions began before AP induction, so their protective effects do not by themselves establish efficacy after disease onset. Nevertheless, these studies show that the gut can contribute to both inflammatory stimulation and protection through the combined effects of microbial products, metabolite signaling, and barrier function.

Acinar injury-derived signals, lipid exposure, oxygen availability, and microbial products together shape the environment encountered by macrophages and neutrophils in AP. These conditions influence both the inflammatory stimuli reaching immune cells and the metabolic processes that support their responses. Their interaction may help explain why immune activity sustains tissue damage in some settings but supports clearance and recovery in others.

## 3 Innate immune metabolic reprogramming

The metabolic stress inputs described above become relevant to disease progression only after they are sensed by immune cells and converted into cellular programs. Macrophages connect local injury signals, inflammatory amplification, and efferocytosis-associated repair, whereas neutrophils contribute to injury propagation through NET formation, ROS production, and glycolysis-linked activation. The central issue is therefore how macrophages and neutrophils translate metabolic stress into distinct metabolic and functional states during AP.

### 3.1 Macrophage metabolic reprogramming

#### 3.1.1 Glycolysis and NAD metabolism in inflammatory activation

Metabolic enzyme regulation links glucose metabolism to macrophage inflammatory function. In cerulein/LPS-induced AP, knockdown of ubiquitin-specific protease 7 (USP7) reduced pancreatic injury, and transfer of USP7-silenced bone marrow-derived macrophages supported a macrophage contribution to this protection. In LPS/IFN-γ-stimulated RAW264.7 cells, USP7 silencing reduced glycolytic acidification, glucose consumption, lactate production, and inflammatory responses, while increasing oxygen consumption. These changes were accompanied by increased pyruvate kinase M2 (PKM2) ubiquitination and reduced Y105 phosphorylation and nuclear translocation (12). The findings link PKM2 modification and localization to both metabolic and inflammatory responses, suggesting that its contribution to macrophage activation involves functions beyond its catalytic role in glycolysis.

Nicotinamide adenine dinucleotide (NAD) salvage metabolism also contributes to macrophage responses to acinar injury. The nicotinamide phosphoribosyl transferase (NAMPT) inhibitor FK866 suppressed inflammatory polarization in bone marrow-derived macrophages exposed to injured acinar cells, whereas the downstream metabolite nicotinamide mononucleotide (NMN) promoted inflammatory polarization. FK866 also reduced pancreatic injury in cerulein-, ethanol/palmitoleic acid-, and taurocholate-induced AP models (47). Although pancreatic NAMPT and NAD levels declined during AP in the same study, the macrophage experiments support a role for NAD salvage metabolism in sustaining inflammatory responses to damaged tissue. Thus, reduced NAD availability in the injured pancreas can coexist with a pro-inflammatory contribution of NAD metabolism in macrophages.

#### 3.1.2 Mitochondrial stress, lipid handling, and macrophage inflammatory responses

Mitochondrial stress can connect danger sensing with macrophage inflammatory responses and cell death. In lung injury associated with cerulein/LPS-induced AP, mtDNA–cGAS–STING signaling was implicated in alveolar and peritoneal macrophage pyroptosis (48). Studies in non-AP inflammatory and sepsis models further link mitochondrial ROS or cGAS–STING signaling to impaired oxidative metabolism and increased glycolysis-related responses in macrophages (49, 50). These findings suggest that mitochondrial dysfunction may influence macrophage responses through both inflammatory signaling and altered energy metabolism. Whether these processes jointly sustain macrophage-mediated injury in AP remains unresolved, but they provide a mechanistic connection between mitochondrial damage and inflammatory activation.

Under lipid-rich conditions, macrophages can both propagate injury and become susceptible to it. Their inflammatory responses and release of acinar-damaging exosomes link fatty acid exposure to injury in neighboring cells (28, 51), whereas CD36-associated ferroptotic changes implicate oxidative damage within macrophages themselves (29). Lipid handling may therefore influence both the damaging activity of macrophages and their ability to survive in inflamed tissue. These effects extend beyond changes in inflammatory polarization, linking macrophage function to cellular vulnerability under lipid stress.

Metabolic enzyme localization offers a further link between lipid sensing and macrophage function. In oxLDL-stimulated macrophages, CD36-dependent mitochondrial translocation of PKM2 promoted mitochondrial ROS production and particle uptake (52). This differs from the association between PKM2 nuclear translocation and inflammatory activation in the USP7 study (12). PKM2 localization links its metabolic role to distinct macrophage functions, with nuclear and mitochondrial redistribution associated with inflammatory activation and ROS production, respectively. The mitochondrial mechanism was demonstrated in an atherogenic setting, but raises the possibility that lipid exposure in AP also alters macrophage function through changes in metabolic enzyme localization.

#### 3.1.3 Metabolic regulation of efferocytosis and tissue repair

Protective macrophage functions extend beyond suppressing inflammation to clearing apoptotic cells and supporting tissue regeneration. In AP, CD47 on injured acinar cells restricts efferocytosis, whereas annexin A1 (Anxa1)-based interventions improve macrophage clearance and reduce pancreatic injury (53, 54). Metabolite signaling can enhance this protective function. Norcholic acid (NorCA) increased apoptotic-cell uptake by bone marrow-derived macrophages and reduced experimental pancreatic injury; PPARα knockdown abolished the increase in uptake (15). This receptor-dependent effect connects metabolite signaling to the removal of dying cells, providing a route through which metabolic regulation can help contain tissue damage.

Macrophages also support regeneration through signals delivered to surviving acinar cells. During experimental AP recovery, lactate promoted repair-associated macrophage gene expression and pancreatic repair (14). Monocyte-derived TREM2-positive macrophages exhibited cholesterol-efflux-related transcriptional features, linking a lipid-associated cell state to regeneration (55). Myeloid Trem2 deletion altered these features, reduced Desert Hedgehog (Dhh) expression, and impaired acinar regeneration, which was partly restored by Hedgehog activation. TREM2-associated macrophage repair therefore involves both lipid-related transcriptional changes and regenerative signaling, although a causal connection between cholesterol efflux and Dhh expression remains unresolved.

Across these studies, metabolic regulation supports distinct macrophage activities rather than a uniform inflammatory or reparative state. Glycolytic enzyme regulation and NAD salvage contribute to inflammatory activation (12, 47), whereas lactate can promote repair-associated responses in a recovery setting (14). Lipid-associated processes likewise accompany macrophage-mediated injury, susceptibility to ferroptosis, and support for acinar regeneration (28, 29, 55). Macrophage contributions to AP progression may therefore depend on how metabolic regulation shapes inflammatory activity, apoptotic-cell clearance, and regenerative signaling within the injured tissue.

### 3.2 Neutrophil metabolism and NET-associated effector programs

Metabolic regulation can support neutrophil inflammatory activation in AP. Exposure to AP mouse serum induced ATP release and increased glycolytic activity in bone marrow-derived neutrophils, whereas extracellular ATP depletion, P2RX1 deficiency, or glycolytic inhibition reduced inflammatory cytokine expression (13). Extracellular ATP provides an activating signal, while glycolysis supports the cellular response. ATP released by responding neutrophils may reinforce this stimulation, providing a possible mechanism for sustaining inflammatory activity.

Patient observations reveal a different metabolic profile. In a cohort of 15 patients, predominantly with mild AP, circulating neutrophils showed lower basal extracellular acidification and reduced PMA-stimulated acidification and oxidative burst compared with healthy controls (56). These results do not establish a temporal transition from activation to exhaustion. Differences in species, cell source, and exposure conditions may contribute to the contrast with AP-serum-stimulated mouse bone marrow neutrophils. Inflammatory stimuli can therefore enhance particular neutrophil responses without producing a uniform increase in glycolytic activity across circulating and tissue-associated populations.

Oxidant production introduces a metabolic requirement beyond ATP generation because NADPH oxidase requires a supply of NADPH. In experimental AP, oxidase deficiency reduced intrapancreatic trypsin activation (57). Phellodendrine treatment reduced p38 MAPK activation, p47phox membrane translocation, ROS production, and NET formation in PMA-stimulated mouse neutrophils, while also reducing NET-associated markers and pancreatic injury in cerulein-induced AP (58). These observations connect oxidase-related responses to pancreatic injury and NET formation, providing a rationale for considering how neutrophil metabolism supplies reducing equivalents for these functions.

The pentose phosphate pathway (PPP) connects glucose utilization to NADPH production. In human neutrophils studied outside AP, fructose maintained ATP levels comparable to those supported by glucose but did not support equivalent NET release after PMA or amyloid fibril stimulation (59). During oxidative burst, recycling glucose carbon through a cyclic PPP supports NADPH regeneration, and disrupting this process impaired NET release (60). Conversely, activation of liver-type phosphofructokinase (PFKL) favored glycolysis, limited PPP engagement, and suppressed NOX2-dependent oxidative burst and NETosis (61). Glucose allocation can therefore separate increased glycolytic activity from the metabolic support required for oxidant production and NET formation.

Substrate availability adds another source of functional variation. In primary human neutrophils, oxidative burst depended strongly on extracellular glucose under the tested conditions, whereas PMA-induced NET release could draw on either extracellular glucose or intracellular glycogen (62). Glycogen may consequently preserve some capacity for NET release when extracellular glucose is limited. Applied to AP, these mechanisms raise the possibility that changes in nutrient availability alter cytokine expression, oxidant production, and NET release to different degrees. The contributions of cyclic PPP activity and glycogen utilization remain to be established directly in AP, but their distinct functional effects offer a mechanistic explanation for why neutrophil-mediated injury may not track glycolytic activity alone.

## 4 Immunometabolites as signaling nodes

Metabolites can connect changes in substrate use to immune responses and tissue injury. They regulate intracellular enzymes and protein modifications, or act through receptors to influence inflammation and clearance. Changes in metabolite abundance during AP can accompany cellular responses without establishing their contribution to tissue injury. **Table 1** summarizes the relevant metabolites and distinguishes findings from experimental AP from mechanisms established outside AP.

**Table 1:** Immunometabolite signaling nodes in AP.

Metabolite/node	Source or AP context	Sensor/target	Target cell or process	Functional output in AP	Evidence boundary	Key refs
Endogenous itaconate/ACOD1	Activated myeloid cells; obesity-related AP	ACOD1-derived itaconate; NRF2-xCT/SLC7A11-GPX4; SDH/NLRP3 mechanisms from basic studies	Acinar anti-ferroptosis defense; myeloid immune regulation	Limits ferroptosis-associated injury and supports antioxidant braking	AP evidence supports ACOD1-associated tissue protection; SDH/NLRP3 mechanisms derive from non-AP studies. Endogenous signaling differs from 4-OI pharmacology.	(16, 63– 66)
4-octyl itaconate (4-OI)	Pharmacologic, cell-permeable itaconate derivative	NRF2-GPX4-related antioxidant and anti-ferroptosis defense	Pancreatic and lung injury compartments	Reduces oxidative injury, inflammatory indices, ferroptosis markers, and AP-associated lung injury	Experimental AP intervention; not identical to endogenous itaconate.	(16, 65, 67)
Lactate: cell-dependent injury and repair	Acinar injury models and experimental AP recovery	H3K18la/NCOA4 in acinar cells; MCT1 and GPR132-associated macrophage responses	Acinar ferritinophagy and ferroptotic injury; macrophage repair-associated transcription	Can aggravate acinar injury or support macrophage-associated pancreatic repair	Effects differ by cell, exposure and model; timing alone does not explain the opposing outcomes.	(14, 68, 69)
Lactate and SCFAs from microbiota	Gut microbiota-derived metabolites after barrier disruption or probiotic intervention	GPR43/FFAR2, HDAC3, or TLR4/NLRP3-related pathways	Macrophage inflammatory responses and intestinal barrier function	Can reduce inflammatory-cell infiltration and inflammatory injury in selected models	Protection varies by metabolite and model; acetate protection was not significant in the high-fat-diet model.	(42–46, 70)
α-ketoglutarate	TCA-cycle intermediate; altered in AP metabolomics	Energy metabolism, NRF2/AMPK-related signaling, dioxygenase-linked biology	Tissue energy stress; candidate immune-regulatory process	Improves pancreatic, lung, and kidney injury in experimental SAP	Energy-protective candidate; immune-cell-intrinsic regulation in AP remains unproven.	(71, 72)
NorCA	Bile acid-related metabolite associated with AP severity	PPARα	Macrophage functional reprogramming and efferocytosis	Promotes macrophage clearance function and reduces AP injury	Non-canonical metabolite signal; direct macrophage metabolic flux change remains undefined.	(15)
Succinate	TCA-cycle intermediate; limited AP metabolomics signal	HIF-1α/IL-1β axis established mainly in non-AP macrophage studies	Candidate macrophage inflammatory signaling	May connect TCA-cycle stress to inflammatory IL-1β expression	Future-validation node, not a confirmed AP amplification metabolite.	(38, 64, 71, 72)

AP, acute pancreatitis; SAP, severe acute pancreatitis; HTG-AP, hypertriglyceridemia-induced acute pancreatitis; SCFAs, short-chain fatty acids; 4-OI, 4-octyl itaconate; NorCA, norcholic acid.

### 4.1 ACOD1 and itaconate-associated antioxidant regulation

Inflammatory activation can induce metabolic responses that restrain inflammation. Aconitate decarboxylase 1 (ACOD1) converts the tricarboxylic acid (TCA) cycle intermediate cis-aconitate into itaconate (63). In activated macrophages studied outside AP, endogenous itaconate inhibits succinate dehydrogenase (SDH), linking altered succinate oxidation to the regulation of inflammatory responses (64). Protein modification provides another route to protection. Studies using the derivative 4-octyl itaconate (4-OI) linked KEAP1 modification to NRF2 stabilization and antioxidant gene expression (65), and NLRP3 modification to reduced interaction with NEK7. Macrophages deficient in itaconate synthesis also showed greater NLRP3 activation (66). Itaconate-associated regulation thus connects inflammatory activation to feedback mechanisms that limit inflammatory effectors and strengthen antioxidant defense.

In AP, ACOD1-associated protection extends to resistance against lipid oxidative injury. ACOD1 was detected predominantly in isolated pancreatic neutrophil and macrophage fractions, while global Acod1 deletion reduced pancreatic NRF2 and glutathione peroxidase 4 (GPX4) protein levels and increased lipid peroxidation and injury. The distribution of ACOD1 raises the possibility that immune-cell metabolism contributes to protection of neighboring pancreatic tissue, although the responsible cellular source and intercellular transfer of itaconate remain unresolved. Acinar cells were responsive to 4-OI: treatment maintained NRF2 and GPX4 and improved survival in erastin-exposed 266–6 cells (16). In experimental AP, 4-OI also reduced lipid peroxidation and pancreatic injury (67). Because GPX4 removes phospholipid hydroperoxides, preservation of this defense offers a plausible explanation for reduced oxidative membrane damage. The genetic and pharmacological results converge on antioxidant protection, without establishing that 4-OI restores endogenous itaconate production.

Obesity may weaken this protective response as lipid stress increases. Pancreatic neutrophils from obese mice with AP had lower ACOD1 abundance alongside reduced abundance of proteins associated with pyruvate metabolism and the TCA cycle; pancreatic NRF2 and xCT/GPX4-associated defenses were also impaired (16). Obesity-associated injury may therefore reflect both greater lipid stress and an inadequate metabolic response to that stress. In these mice, 4-OI improved pancreatic redox measures and reduced tissue injury. The persistence of this pharmacological response suggests that impaired endogenous protection can coexist with an antioxidant system that remains responsive to intervention.

### 4.2 Lactate as a context-dependent bidirectional signal

Lactate can aggravate acinar injury or support macrophage-mediated protection in experimental AP. In L-arginine-induced rat pancreatitis and cerulein-stimulated AR42J cells, lactate increased NCOA4 expression and ferritinophagy-associated ferroptotic injury, accompanied by enrichment of histone H3 lysine 18 lactylation (H3K18la) at the NCOA4 promoter. NCOA4 knockdown attenuated lactate-aggravated damage (68). Lactate-derived histone lactylation provides a mechanism linking cellular metabolism to gene transcription (69). By contrast, lactate administration during experimental AP recovery increased macrophage lactylation and repair-associated gene expression while improving pancreatic recovery (14). These opposing effects link lactate to different cellular processes, including iron mobilization in injured acinar cells and repair-associated responses in macrophages.

Macrophage responses involve both intracellular lactate handling and receptor-associated signaling. In RAW264.7 cells exposed to conditioned medium from injured acinar cells, lactate increased H3K18la and expression of Lrg1, Vegfa, and Retnla. The monocarboxylate transporter 1 (MCT1) inhibitor AZD3965 attenuated these changes, supporting a contribution of lactate transport to lactylation and repair-associated transcription (14). In the same experimental system, a GPR132 antagonist reversed lactate-mediated suppression of JAK2–STAT1 signaling and pro-inflammatory gene expression. Lactate transport and receptor-associated signaling may therefore provide complementary routes for promoting repair-associated responses and limiting macrophage inflammatory activation.

Protective effects are also evident outside the recovery setting. In a study of Bifidobacterium-associated protection, oral lactate administered before AP induction reduced pancreatic injury. Complementary experiments in peritoneal macrophages showed suppression of LPS-induced inflammatory responses involving TLR4/MyD88–NF-κB and NLRP3/caspase-1 signaling (70). Protection occurred without an increase in the proportion of pancreatic CD206-positive macrophages, indicating that reduced inflammation need not coincide with expansion of this marker-defined population. The injury-promoting study also administered lactate before AP induction, but used intraperitoneal delivery and a different disease model (68). Timing alone therefore does not explain the opposing outcomes; differences in exposure and cellular responses offer additional explanations.

Lactate thus connects glycolytic metabolism to processes with opposing consequences for pancreatic injury. Enhanced ferritinophagy can increase acinar susceptibility to lipid oxidative damage, whereas suppression of macrophage inflammation and support for repair can favor recovery. The relative contribution of these responses may determine whether lactate aggravates injury or supports its resolution.

### 4.3 α-KG in energy protection and potential immune regulation

TCA-cycle intermediates can influence both tissue energy metabolism and inflammatory signaling in AP. Clinical metabolomics studies identified altered α-ketoglutarate (α-KG) abundance (71), while supplementation beginning before AP induction reduced pancreatic and distant-organ injury in cerulein/LPS-treated mice (72). Protection was accompanied by increased pancreatic ATP, improved mitochondrial ultrastructure, and recovery of several respiratory-chain proteins and NRF2- and AMPK-related measures. The coexistence of elevated α-KG and impaired energy metabolism indicates that metabolite abundance alone does not describe its effective utilization. Supplementation may support tissue recovery despite endogenous accumulation, although the protective response has not been assigned specifically to macrophages or neutrophils.

The effects of α-KG also extend to regulation of inflammatory gene expression. In non-AP endotoxemia research, the cell-permeable derivative dimethyl-α-KG worsened LPS-associated injury, with macrophage experiments linking this response to TET2-mediated AIM2 regulation and PANoptosis (73). This differs from the protection observed with α-KG supplementation in experimental AP. The contrast raises the possibility that support for energy metabolism and regulation of inflammatory genes produce different outcomes depending on the cellular setting. Compound formulation, stimulus, and target-cell differences prevent the two interventions from being treated as equivalent.

Succinate provides a further connection between TCA-cycle metabolism and inflammatory signaling. Pancreatic succinate abundance increased in mice with AP, while non-AP macrophage experiments linked intracellular succinate accumulation to HIF-1α-dependent IL-1β expression (38, 72). These observations raise the possibility that altered succinate metabolism contributes to inflammation in AP as well as accompanying tissue energy stress. Such a role would depend on succinate accumulation or signaling within responsive cells, which whole-pancreas measurements do not resolve. Alongside the α-KG studies, this potential mechanism places TCA-cycle intermediates at the intersection of mitochondrial energy metabolism and immune regulation, with consequences that depend on how and where each metabolite acts.

### 4.4 Functional regulation by bile acid-related metabolites and short-chain fatty acids

Metabolites can regulate macrophage function through signaling mechanisms beyond their role as metabolic substrates. NorCA was elevated in the circulation of patients and mice with severe AP, while supplementation reduced experimental pancreatic injury and enhanced apoptotic-cell uptake by bone marrow-derived macrophages (15). PPARα inhibition or knockdown abolished the increase in uptake, linking this clearance response to receptor-dependent regulation. The coexistence of elevated NorCA and benefit from supplementation is compatible with a protective response that remains insufficient to contain injury, although endogenous compensation has not been established. PPARα dependence identifies a signaling requirement without establishing direct NorCA binding or increased fatty acid oxidation as the mechanism.

Short-chain fatty acids influence macrophage inflammation through different receptor-associated and transcriptional mechanisms. Acetate suppressed macrophage inflammatory responses through GPR43/FFAR2-associated signaling in experimental AP (44), whereas butyrate-related protection involved HDAC3 inhibition and altered histone acetylation in a high-fat-diet AP model (46). These cellular effects do not translate uniformly across disease settings. Acetate reduced injury in a cerulein model but did not significantly improve injury in the separate high-fat-diet study. Butyrate also improved intestinal barrier measures in experimental AP, indicating that its protective effects may combine altered macrophage responses with reduced exposure to gut-derived inflammatory stimuli (45). Differences in model and treatment conditions may therefore influence how receptor signaling, transcriptional regulation, and barrier protection contribute to the overall tissue response.

Across these metabolite classes, metabolic regulation connects immune-cell activity with the susceptibility of pancreatic tissue to injury. Antioxidant defense and apoptotic-cell clearance can help contain damage, while changes in inflammatory transcription and regulated cell death can sustain or aggravate it. A metabolite may influence several of these processes, so its contribution to AP progression depends on the cells it reaches and the functions it modifies. This functional diversity links immunometabolite signaling to both inflammatory amplification and endogenous protection.

## 5 Metabolism-driven amplification programs and endogenous braking programs

The consequences of pancreatic injury depend on both immune effector activity and the susceptibility of surrounding tissue to damage. Metabolic regulation may alter this interaction by sustaining inflammatory functions, modifying acinar antioxidant defense, or supporting the removal of apoptotic cells. These processes may affect how much new injury occurs and how long damaged tissue continues to stimulate immune cells. The amplification-braking interface is summarized in **Figure 2**.

**Figure 2:**
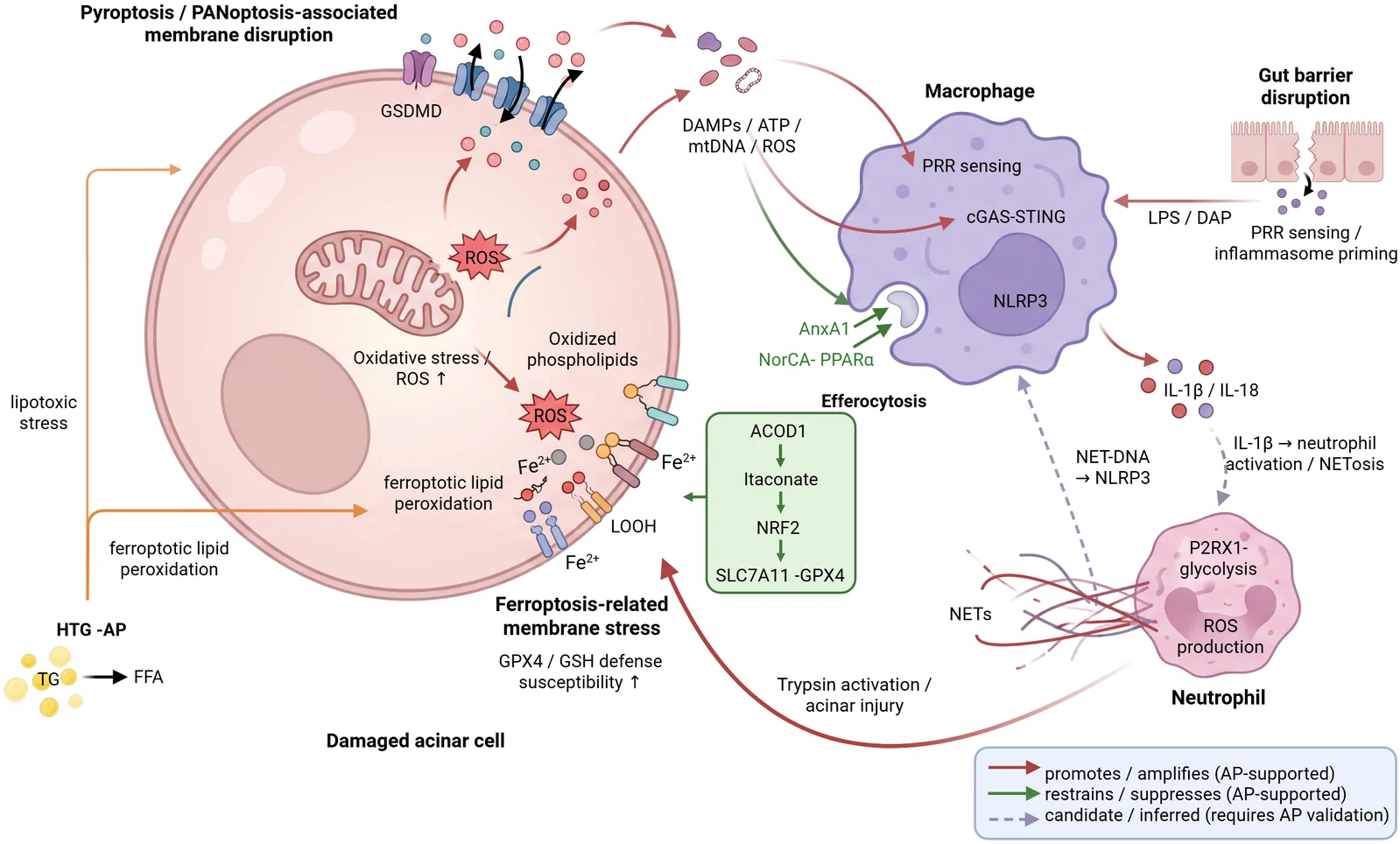
Amplification-braking interface in AP immunometabolism. Damaged acinar cells release DAMPs, ATP, mtDNA, ROS, and lipid peroxidation-related signals that promote macrophage PRR sensing, cGAS-STING signaling, and NLRP3-associated inflammatory signaling. Neutrophil activation and NET formation can further amplify acinar injury, while regulated cell-death programs connect pyroptosis/PANoptosis-associated membrane disruption with ferroptosis-related lipid peroxidation and GPX4-GSH defense failure. In parallel, efferocytosis-competent macrophages, AnxA1, NorCA-PPARα, and ACOD1/itaconate-NRF2-SLC7A11-GPX4 signaling represent endogenous braking outputs that may reduce DAMP substrate and restrain ferroptosis-associated injury. Red arrows indicate AP-supported promoting or amplifying relationships; green arrows indicate AP-supported restraining relationships; dashed purple arrows indicate candidate or inferred links requiring AP validation. DAMPs, damage-associated molecular patterns; mtDNA, mitochondrial DNA; PRR, pattern-recognition receptor; NETs, neutrophil extracellular traps; GPX4, glutathione peroxidase 4; GSH, glutathione; AnxA1, Annexin A1. Created with Biorender.com.

### 5.1 Metabolism-supported inflammatory-death amplification programs

Injured acinar cells release signals that activate macrophages and recruit neutrophils. Metabolic regulation can sustain the resulting inflammatory activity, as illustrated by the association of USP7-dependent PKM2 regulation with macrophage glycolytic remodeling and cytokine expression in AP (12). NLRP3-dependent cytokine responses can propagate inflammation between immune cells (8), while XO-associated glucose metabolism contributes to injury in acinar-cell experiments (37). Metabolic changes may therefore reinforce inflammation from both sides of the interaction, supporting immune effector activity while increasing the vulnerability of the tissue exposed to it.

Further acinar injury can renew the stimuli driving this response. The inflammatory consequences of cell death depend partly on the release of cellular contents (74). In cerulein-induced AP, ZBP1 inhibition reduced pancreatic injury together with markers associated with pyroptosis, apoptosis, and necroptosis (75). These results implicate multiple death-related processes without establishing their integration through an assembled PANoptosome. Where cell death involves membrane rupture, released intracellular material can provide additional stimulation to recruited immune cells, allowing inflammation to persist beyond the initiating insult.

NETs provide a direct route from neutrophil activity back to acinar damage. DNase I treatment reduced pancreatic injury and neutrophil infiltration in experimental AP, while NET preparations and histones induced trypsin activation in isolated acinar cells (6). PAD4-deficient mice provide complementary evidence linking NET formation to AP severity (76). Neutrophils recruited by tissue injury can thus generate effectors that aggravate that injury. Non-AP studies of cyclic PPP activity and PFKL regulation offer a metabolic explanation for how NET formation may be sustained, although this dependence has not been established directly in AP (60).

NETs may also propagate inflammation through macrophage cytokine release. NET preparations from HMGB1-stimulated neutrophils increased IL-1β release from HMGB1-treated THP-1-derived macrophages (77), and NET-DNA elicited NLRP3-associated responses under LPS-primed conditions in adult-onset Still’s disease research (78). A possible return signal emerges from abdominal aortic aneurysm studies, in which IL-1β promoted NET formation through a mechanism involving CerS6 and C16 ceramide (79). If these responses coexist in AP, ceramide synthesis could connect macrophage-derived IL-1β to further NET formation, creating a metabolically supported feedback loop between the two cell populations.

Lipid peroxidation can reinforce amplification by increasing cellular susceptibility to injury. In experimental AP, wedelolactone reduced pancreatic injury alongside ferroptosis- and pyroptosis-related changes, and GPX4 knockdown weakened its protection in cerulein-treated AR42J acinar cells (80). Preservation of GPX4-mediated phospholipid peroxide removal may therefore limit acinar damage and the inflammatory stimuli released from injured tissue. Related mechanisms operate in myeloid cells outside AP. Increased labile iron promoted NLRP3-dependent IL-1β release from LPS-primed human monocytes (81), while macrophage GPX4 loss promoted lipid peroxidation and GSDMD-mediated pyroptosis in sepsis research (82). These studies raise the possibility that impaired control of lipid oxidation intensifies both tissue injury and myeloid inflammatory responses, although the myeloid mechanisms remain to be established in AP.

Inflammatory amplification may consequently persist when metabolic support for immune effector activity coincides with continued generation of injury-derived stimuli. Gut barrier disruption can add microbial products to this stimulation (40). Effective clearance and antioxidant protection could limit the renewal of these signals, providing a functional counterweight to the processes that sustain damage.

### 5.2 Metabolism-dependent endogenous braking programs

Efficient removal of apoptotic cells can limit the persistence of injury-derived inflammatory stimuli. In cerulein-induced AP, Anxa1 deficiency increased acinar cell death and pancreatic injury, while Anxa1-deficient macrophages showed impaired efferocytosis. The same study identified acinar-derived cGAMP as a stimulus for macrophage STING activation; Anxa1 mRNA delivery improved clearance and reduced STING-associated inflammation and injury (54). These results connect the fate of dying acinar cells to subsequent macrophage activation. Additional AP studies implicate Axl/MerTK in tissue repair and show that TGM2 can impair macrophage efferocytosis through inhibition of STAT6–GAS6 signaling (83, 84). Engulfment can also sustain inflammation when macrophages take up zymogen-containing acinar material. In experimental pancreatitis, cathepsin B-dependent trypsinogen activation within macrophages promoted inflammatory cytokine production, indicating that the consequences of uptake depend on the cargo and its intracellular processing (85).

Metabolic regulation can strengthen this clearance response and influence how macrophages respond to engulfed material. NorCA enhanced macrophage efferocytosis through PPARα-associated signaling, and its protection in experimental AP was sensitive to PPARα antagonism (15). Conversely, engulfment itself supplies metabolites that can modify macrophage function. In non-AP experiments, fatty acids derived from apoptotic cells supported mitochondrial metabolism and NAD-linked signaling that promoted IL-10 production and tissue repair (86). Clearance may therefore restrain inflammation in two connected ways: removing dying cells before they generate further inflammatory stimuli and using their metabolic cargo to support protective macrophage responses.

Antioxidant defense complements clearance by limiting new cellular injury. In stressed acinar cells, xCT-dependent cystine transport maintains glutathione availability and limits lipid peroxidation, linking cellular resistance to oxidative injury with substrate supply (87). Acod1 loss increased pancreatic lipid peroxidation and injury, whereas 4-OI treatment supported NRF2/GPX4-associated protection in experimental AP (16, 67). Myeloid ACOD1 expression provides a possible link between immune-cell metabolism and tissue protection, although the responsible cellular source and transfer of endogenous itaconate remain unresolved. In non-AP sepsis-associated lung injury research, 4-OI also reduced ferroptosis-related changes in macrophage models (88), raising the possibility that antioxidant regulation protects both injured tissue and responding immune cells. Limiting new oxidative damage while removing existing apoptotic cells could reduce both the generation and persistence of inflammatory stimuli.

Gut-derived metabolites may further restrict inflammation by acting on barrier function and macrophage responsiveness. Acetate- and butyrate-related studies support modulation of macrophage inflammatory signaling, while butyrate and inulin interventions also improved measures of intestinal barrier integrity. These effects could reduce exposure to microbial products while limiting the inflammatory response to them, although protection varies across AP models (44–46).

Clearance, antioxidant defense, and barrier-associated protection thus act at different points in the processes that sustain inflammation. Their combined activity may reduce continuing immune stimulation even while pancreatic injury is still occurring, providing a functional basis for endogenous braking.

### 5.3 A functional framework for amplification and braking

We propose an integrated amplification-braking framework linking macrophage and neutrophil immunometabolism to the persistence or containment of pancreatic injury. Amplification occurs when metabolically supported inflammatory effectors aggravate tissue damage and renew the signals that stimulate immune cells. Braking comprises clearance, antioxidant protection, and repair-associated functions supported by metabolic adaptation and metabolite signaling. These functions can operate concurrently within the injured pancreas. Acinar injury connects them by acting both as a source of immune stimulation and as a consequence of inflammatory effector activity.

The amplification arm links metabolic support for immune activation to the continued generation of tissue-derived stimuli. AP studies connect macrophage and neutrophil glycolytic regulation to inflammatory responses (12, 13), while separate experiments establish contributions of NETs and oxidant production to pancreatic injury (6, 57, 76). Together, these processes provide a plausible route by which an immune response initiated by acinar damage could help sustain that damage. The metabolic and effector components have not, however, been demonstrated as a complete feedback circuit in AP; P2RX1-associated glycolysis, for example, has not been directly linked to NET-mediated tissue injury.

Braking acts at complementary points in this interaction. Efferocytosis removes apoptotic cells that could otherwise contribute to continued inflammatory stimulation, whereas antioxidant defense limits new oxidative injury and the generation of additional danger signals (15, 16, 54). Repair-associated macrophage responses can further support recovery of surviving tissue (14, 55). These protective functions need not strengthen in parallel with inflammatory activation. In obese mice with AP, inflammatory infiltration coexists with reduced neutrophil ACOD1 abundance and impaired pancreatic antioxidant defenses (16). Increased immune-cell recruitment may therefore coexist with inadequate metabolic support for tissue protection. This provides one explanation for persistent injury despite the presence of endogenous protective responses.

The framework builds on experimental evidence that SIRS and CARS can coexist in AP (8), while focusing on the metabolic support for specific inflammatory and protective functions. Protective braking is distinct from generalized immunosuppression: reduced immune responsiveness may compromise host defense without improving apoptotic-cell clearance, antioxidant protection, or tissue repair. Its defining feature is therefore containment of damage rather than reduced inflammatory activity alone. Whether differences in metabolic support for amplification and braking help explain divergent AP courses remains a testable proposition. Longitudinal assessment of inflammatory effectors alongside clearance and antioxidant function, as discussed in Section 7.2, could examine this relationship.

## 6 Therapeutic implications of the amplification-braking framework

Persistent immune-mediated injury and inadequate clearance or antioxidant protection may coexist during AP. Limiting amplification and restoring braking are therefore potentially complementary therapeutic goals, and time from onset alone cannot determine which is needed. HTG-AP also involves an upstream metabolic burden that may sustain lipotoxic exposure.

### 6.1 Targeting immunometabolic support for inflammatory amplification

Metabolic processes that support leukocyte activation offer potential targets for limiting persistent pancreatic injury. USP7 knockdown reduced injury in cerulein/LPS-induced AP, while macrophage experiments linked this intervention to reduced glycolytic activity and inflammatory cytokine expression (12). Disruption of P2RX1 signaling also reduced experimental AP injury, and P2RX1 deficiency or glycolytic inhibition suppressed inflammatory cytokine expression in neutrophils exposed to AP serum (13). These results provide a rationale for targeting the metabolic support of inflammatory responses, although the cellular metabolic measurements do not fully establish the mechanism of protection *in vivo*.

The direction of intervention depends on the function of the enzyme being targeted. In the USP7 study, the PKM2 inhibitor Compound 3K partially reversed the protection associated with USP7 knockdown (12). Conversely, the PKM2 tetramer-promoting compounds TEPP-46 and DASA-58 reduced HIF-1α-associated IL-1β responses in LPS-stimulated macrophages outside AP (89). Neutrophil experiments provide another example: PFKL activation by NA-11 favored glycolysis, restricted PPP-derived NADPH supply, and suppressed NOX2-dependent oxidative burst and NETosis (61). Selective changes in enzyme activity or substrate allocation may therefore limit particular inflammatory functions without requiring a general reduction in glycolysis.

A single intervention may also combine reduced inflammatory activity with enhanced antioxidant protection. Pretreatment with KAN0438757, a PFKFB3 inhibitor, reduced experimental AP injury and lowered inflammatory cytokines and ROS in stimulated macrophages. HO-1 loss or inhibition supported a contribution of HO-1 to protection (90). These effects connect suppression of inflammatory activity with antioxidant defense, although neither PFKFB3-specific action nor altered glycolytic flux was established as the mechanism of protection. Such combined effects provide a pharmacological basis for exploring interventions that limit amplification while supporting protective functions.

Limiting further effector activity and removing already released injury mediators are potentially complementary approaches. DNase I reduced experimental AP injury by degrading extracellular DNA (6), while NAC combined with DNase I provided evidence for combining reduced NET formation with removal of released material (91). Inhaled recombinant human DNase has demonstrated clinical benefit in cystic fibrosis (92), but this experience does not establish the efficacy or safety of systemic administration in AP. NLRP3 inhibition, Gsdmd deletion, and separate disulfiram experiments also support targeting downstream inflammatory or cell-death processes (93–95). Disulfiram directly inhibited GSDMD pore formation in non-AP experiments (96), providing a mechanistic rationale for its study in pancreatitis. These findings justify testing combinations with metabolic interventions, but do not yet establish their added benefit or optimal sequence in AP.

### 6.2 Enhancing endogenous braking programs

Enhancing macrophage clearance offers a direct route to curtailing the persistence of injury-derived inflammatory cues. In preclinical AP, NorCA enhanced efferocytosis via PPARα-dependent signaling to attenuate disease severity (15), while lipid-nanoparticle-mediated delivery of Anxa1 mRNA accelerated apoptotic clearance and attenuated STING-associated inflammatory signaling (54). Although this Anxa1 strategy establishes clearance as a viable therapeutic target, it does not demonstrate cell-intrinsic metabolic reprogramming. Engulfment itself, however, imposes distinct bioenergetic demands. In non-AP settings, fatty acids derived from apoptotic corpses fuel mitochondrial respiration and NAD-linked cascades to promote IL-10 synthesis and tissue repair (86). Consequently, clearance-directed therapies may benefit not merely from increasing target uptake, but from preserving the metabolic machinery that processes engulfed material.

While clearance eliminates cells that are already dying, antioxidant support complements this process by shielding surviving tissue from lethal injury. In erastin-stressed acinar cells, the cell-permeable itaconate derivative 4-OI preserved NRF2/GPX4-associated defenses, whereas *in vivo* pretreatment limits pancreatic necrosis and associated lung injury in experimental AP (16, 67). These benefits validate the therapeutic tractability of this pathway, yet derivative-mediated protection cannot be conflated with the functional restoration of endogenous myeloid itaconate synthesis. Notably, pancreatic neutrophils from obese AP mice exhibit marked ACOD1 depletion alongside collapsed antioxidant capacity (16). This failure of endogenous braking suggests that pharmacological reinforcement may be especially valuable in metabolically compromised hosts—a therapeutic proposition that warrants systematic comparison across distinct metabolic backgrounds.

Because active cell death continuously generates fresh debris for clearance, protective interventions may retain utility while tissue destruction is actively underway. Indeed, both NorCA (administered 30 minutes post-induction) and Anxa1 mRNA carriers (delivered after the initial cerulein challenge) conferred marked protection during active disease onset (15, 54). By contrast, *in vivo* proof-of-concept for 4-OI relied strictly on pre-injury administration (67). While these experimental models demonstrate that braking pathways can be therapeutically engaged during active injury induction, they do not yet define a clinically viable post-onset treatment window.

Critically, augmenting tissue protection must be distinguished from precipitating systemic immune paralysis. Persistently low monocytic HLA-DR expression reliably marks adverse clinical outcomes in patients with AP (97), yet this association does not imply that reinforcing clearance or antioxidant defenses inevitably compromises host defense. The ultimate therapeutic objective is twofold: to extinguish persistent danger signals at the injury site while preserving systemic antimicrobial vigilance. Validating whether candidate interventions achieve this balance will require evaluating their effects on both sterile tissue destruction and pathogen control.

### 6.3 Targeting lipid burden and downstream injury in HTG-AP

Lowering triglycerides may reduce the substrate available for lipotoxic injury in HTG-AP, but circulating concentrations do not directly measure pancreatic lipid exposure. At equivalent triglyceride concentrations, lipoproteins from different experimental sources released different amounts of free fatty acids and caused different degrees of acinar injury (98). Substrate reduction may therefore limit further damage without immediately terminating inflammation already sustained by injured tissue.

Clinical evidence likewise separates triglyceride reduction from patient benefit. A randomized trial of 22 patients with a non-severe prognosis found no significant difference between plasma exchange and insulin in 24-hour triglyceride reduction or disease severity; its size and biochemical primary endpoint preclude conclusions of equivalence in organ outcomes (99). Observational analyses reported improved attainment of some triglyceride targets with therapeutic plasma exchange (TPE), without consistent mortality or organ-outcome benefits (100, 101). The 2023 ASFA guidelines classify TPE/lipoprotein apheresis for severe HTG-AP as Category III, Grade 1C, indicating an undetermined optimal role and the need for individualized decisions (102). The uncertain relationship between faster triglyceride reduction and clinical recovery provides a rationale for also targeting the tissue injury sustained downstream of lipid exposure.

Limiting lipid peroxidation may complement substrate reduction. Ferrostatin-1 reduced oxidative injury in poloxamer-407/cerulein-induced HTG-AP mice (103), while liproxstatin-1 pretreatment improved GPX4/xCT-associated defense and pancreatic injury in high-fat-diet/cerulein-treated rats (104). These interventions could strengthen antioxidant braking by reducing new tissue damage and renewed immune stimulation. However, the tissue-level results do not establish immune-cell metabolic targeting, efficacy after disease onset, or superiority of combined treatment over lipid lowering alone.

Therapeutic strategies emerging from this framework address complementary aspects of AP: metabolic support for inflammatory effectors, clearance and antioxidant protection, and lipid-associated tissue injury. Their clinical value will depend on benefit after disease onset while preserving host defense. **Table 2** summarizes the evidence and limitations for candidate interventions, and **Figure 3** outlines their translational status and potential roles in limiting amplification or supporting protective functions.

**Table 2:** Translational intervention hierarchy.

Strategy	Targeted program	Etiology/stage	Evidence maturity	Potential use	Main limitation or risk	Key refs
TG lowering: insulin or TPE	Upstream lipotoxic substrate pressure	HTG-AP; early and progressive phases	Human AP evidence for TG reduction; clinical endpoint benefit uncertain	Rapid reduction of circulating TG burden in selected HTG-AP patients	TG lowering does not reliably translate into lower organ failure or mortality; TPE remains context-dependent.	(98–102)
DNase I, PAD4-related targeting, chloroquine, NAC + DNase I	NET burden or NET formation	Experimental AP/SAP; treatment timing varies	Experimental AP evidence; inhaled recombinant human DNase has clinical efficacy in cystic fibrosis.	Limit neutrophil effector amplification and NET-derived injury inputs	Prior clinical use of inhaled DNase I does not validate systemic delivery in AP; dosing, timing and host-defense effects remain uncertain.	(6, 76, 91, 92)
NLRP3 inhibition	Inflammasome-cytokine arm	Experimental AP; MCC950 given with induction	Experimental AP evidence; clinical readiness varies by compound	Reduce IL-1β/IL-18-associated inflammatory injury	Global inflammasome inhibition may impair antimicrobial defense in infection-risk stages.	(23, 93)
GSDMD-related targeting and disulfiram	Pyroptotic pore formation/inflammatory death output	Early to progressive AP injury	Experimental AP evidence; prior human use of disulfiram does not establish AP efficacy	Limit pyroptosis-associated cytokine release and membrane pore injury	Disulfiram is multi-target; protection does not establish GSDMD dependence. AP-specific safety and treatment windows require validation.	(94–96)
Ferrostatin-1, liproxstatin-1, iron/redox modulation	Ferroptosis-associated lipid peroxidation	HTG-AP, obesity-related SAP, redox-stress-dominant AP	Experimental AP or HTG-AP evidence	Limit lipid ROS, GPX4-GSH failure, and ferroptosis-related injury	Mostly preclinical; biomarkers and patient selection are not established.	(80, 103, 104)
Annexin A1/AnxA1 mRNA liposomes	Macrophage efferocytosis and resolution	Experimental AP; administration after first cerulein injection	Experimental AP evidence	Promote apoptotic-cell clearance and reduce persistent danger signaling	Early experimental dosing does not establish a clinical rescue window; direct metabolic reprogramming was not demonstrated.	(54)
NorCA–PPARα	Metabolite-nuclear receptor support of efferocytosis	Experimental AP; 30 min after first cerulein injection	Experimental AP evidence	Enhance macrophage clearance function and repair-associated output	Direct immune-cell metabolic flux mechanism remains to be defined.	(15)
4-OI or itaconate-axis enhancement	NRF2-xCT/SLC7A11-GPX4 braking	Obesity-related AP; ferroptosis-associated injury	Experimental AP evidence plus basic immunometabolism	Restore antioxidant and anti-ferroptosis defense	4-OI is a derivative; macrophage ferroptosis mechanisms also draw on non-AP sepsis evidence. Treatment windows require validation.	(16, 64–67, 88)
Succinate/SDH and α-KG-related modulation	TCA-cycle immunometabolite signaling	Candidate metabolic-stress states	Mainly non-AP mechanism or experimental tissue-protection evidence	Potentially tune HIF-1α, IL-1β, energy stress, or epigenetic regulation	Not ready as AP immune-cell-targeted therapy without direct validation.	(38, 64, 72, 73)

TG, triglycerides; TPE, therapeutic plasma exchange; NETs, neutrophil extracellular traps; NAC, N-acetylcysteine; 4-OI, 4-octyl itaconate; α-KG, α-ketoglutarate. Experimental protection does not establish clinical efficacy in AP; proposed disease phases are not validated treatment windows.

**Figure 3:**
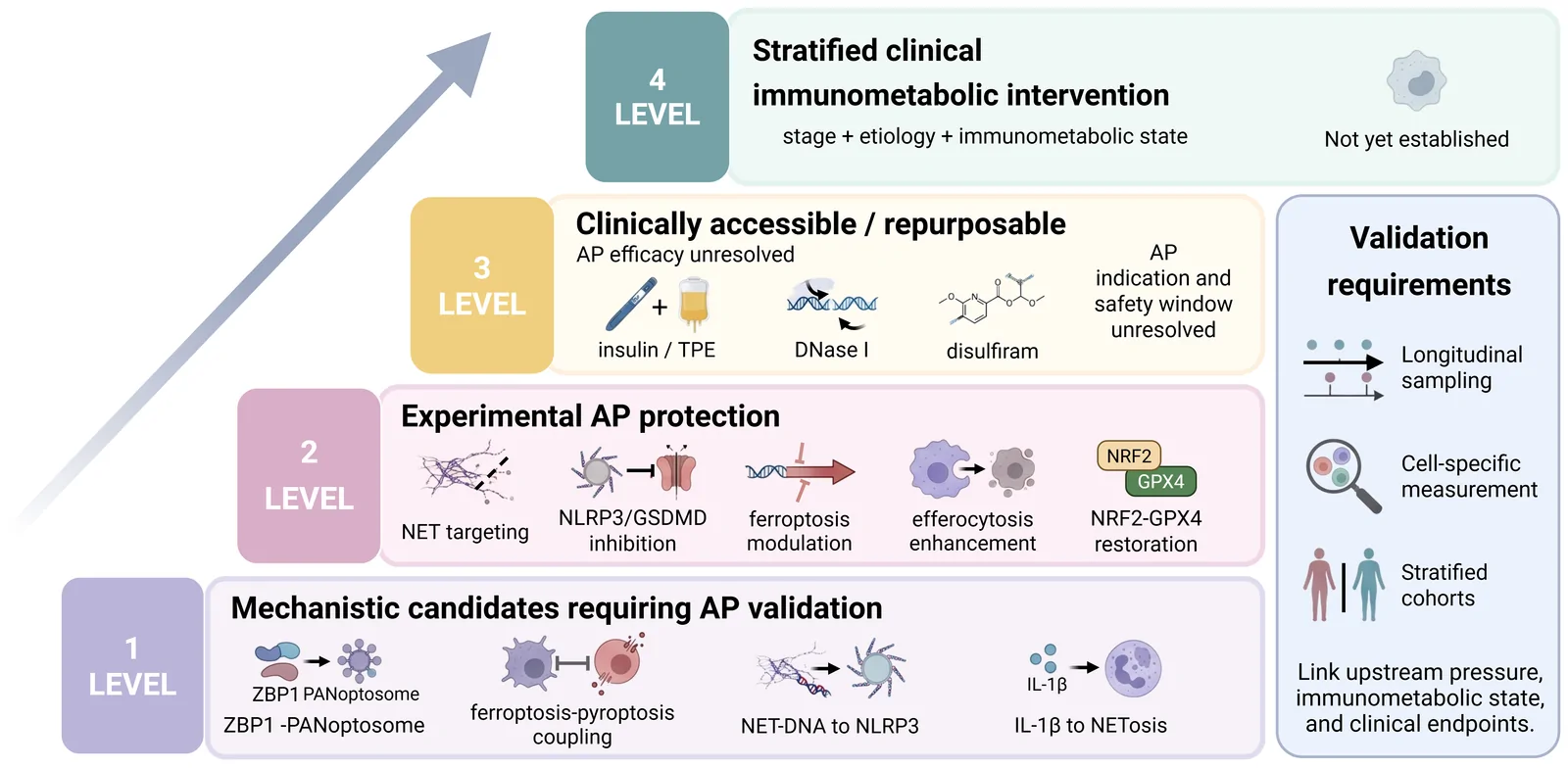
Translational maturity of immunometabolic interventions in acute pancreatitis. Candidate immunometabolic interventions are organized by translational maturity rather than by mechanistic plausibility alone. Level 1 includes mechanistic candidates that still require AP-specific validation, such as ZBP1-PANoptosome signaling, ferroptosis-pyroptosis coupling, NET-DNA-to-NLRP3 signaling, and IL-1β-to-NETosis feedback. Level 2 includes interventions with protective effects in experimental AP models, including NET targeting, NLRP3/GSDMD inhibition, ferroptosis modulation, efferocytosis enhancement, and NRF2-GPX4 restoration. Level 3 includes clinically accessible or potentially repurposable approaches, such as insulin/TPE, DNase I, and disulfiram, for which AP-specific efficacy, indication, and safety windows remain unresolved. Level 4 represents the proposed goal of stratified clinical immunometabolic intervention based on disease stage, etiology, and immunometabolic state, which has not yet been established. Validation requires longitudinal sampling, cell-specific measurement, and clinically stratified cohorts linking upstream pressure, immunometabolic state, and clinical endpoints. AP, acute pancreatitis; TPE, therapeutic plasma exchange; NETs, neutrophil extracellular traps; GSDMD, gasdermin D. Created with Biorender.com.

## 7 Future directions and conclusions

Whether metabolic regulation alters the course of AP depends on how its effects on specific immune functions translate into tissue injury. Existing studies support several of these links, but differences in cellular compartment, evidence source, and observation period limit their integration into an explanation of disease progression.

### 7.1 Evidence boundaries of the current framework

The evidence base has several boundaries. First, the proposed balance between inflammatory-death amplification and endogenous braking has not been quantitatively validated in AP patients or animal models using simultaneous longitudinal measurements. Second, several links in the amplification network still depend mainly on non-AP inflammatory models, including NET-derived DNA and macrophage NLRP3, IL-1β and NETosis, Fe^2+^/ROS and NLRP3, and GSDMD pores and the system xc^-^/GPX4 axis. Third, most therapeutic directions are still supported by experimental AP studies or mechanistic plausibility, whereas clinical treatment windows, safety, and infection risk are unresolved. Experimental models reproduce different components of AP, so mechanistic findings and treatment responses require interpretation in relation to the induction method and injury severity (105).

The scope of this review is also deliberately limited. We focused on myeloid innate immune cells, particularly macrophages and neutrophils, and did not systematically address adaptive immunity, sex differences, genetic background, comorbidities, or progression from AP to chronic pancreatic disease. These factors may modify AP immunometabolic trajectories, but they require separate analysis. The amplification-braking framework should therefore be viewed as a testable model, not as a mature clinical classification system.

### 7.2 Linking metabolic changes to amplification and braking functions

Testing the amplification-braking framework requires connecting metabolic changes with the cellular functions that sustain or contain injury. Lower circulating cytokine concentrations may accompany either reduced tissue damage or impaired immune responsiveness, while increased ACOD1 or GPX4 expression may coexist with ongoing lipid peroxidation. Paired metabolic, functional, and injury measurements could distinguish these possibilities. **Box 1** outlines candidate readouts for examining these relationships rather than a validated clinical panel.

Existing studies provide starting points for this approach. Patient leukocyte bioenergetics reveal differences between cell populations and their responses to stimulation (56), while AP experiments connect apoptotic-cell uptake and antioxidant protection with tissue outcomes (15, 16, 54). A central question is whether impaired clearance or persistent lipid peroxidation identifies delayed recovery among patients with comparable initial severity and inflammatory activity. Such an association would support evaluating the additional prognostic value of protective functions. For amplification, experimental studies could test whether altered neutrophil glycolysis or PPP activity changes NET release and subsequent acinar injury, directly linking the metabolic mechanisms discussed in Section 3.2 to their proposed tissue consequences.

Longitudinal assessment is needed because metabolic activity, immune function, and tissue injury may change at different rates. Sampling should document time from symptom onset, admission, and treatment, while accounting for infection, metabolic comorbidities, and interventions that alter measured concentrations or cell responses. Circulating triglycerides and free fatty acids describe aspects of lipid exposure, whereas intracellular flux requires separate assessment. Blood-cell measurements are accessible but may differ from the responses of immune cells within injured pancreatic tissue.

Experimental perturbation can then test the mechanisms suggested by these associations. If metabolic treatment improves both a cellular function and pancreatic injury, selectively disrupting that function can determine whether it contributes to protection. Persistence of benefit would instead point to additional mechanisms. Combining longitudinal clinical observations with such functional tests would clarify which metabolic changes participate in amplification or braking and which primarily accompany the disease course.

### 7.3 Conclusions

Experimental and translational evidence in acute pancreatitis increasingly demonstrates that metabolic adaptations do not merely reflect leukocyte activation, but actively instruct the cellular effectors that drive tissue damage or enforce its containment. The amplification-braking framework conceptualizes these intertwined dynamics by proposing that progressive injury is sustained when metabolically primed inflammatory programs perpetuate parenchymal destruction, while endogenous clearance, antioxidant defenses, and barrier protections prove insufficient to extinguish injury-derived stimuli. Crucially, this perspective moves beyond rigid classifications that assign permanent damaging or protective phenotypes to specific immune lineages or metabolic pathways, emphasizing instead the context-dependent functional outputs delivered at the acinar–leukocyte interface.

For prospective immunometabolic therapeutics, the central translational challenge is not the indiscriminate suppression of cellular bioenergetics, but the selective restriction of cytotoxic effector programs while safeguarding the clearance, antioxidant reserves, and antimicrobial surveillance necessary for tissue restitution and host defense. Moving from preclinical target validation to clinical utility requires longitudinal, paired assessments that couple intracellular metabolic fluxes to verifiable cellular execution and hard organ outcomes. While this framework provides a mechanistic foundation for deciphering immunometabolic checkpoints and prioritizing candidate targets, it represents a structured conceptual paradigm for hypothesis testing rather than an established clinical stratification or prescribing algorithm for patients with acute pancreatitis.
